# Mini Inside-Out Nuclear Magnetic Resonance Sensor Design for Soil Moisture Measurements

**DOI:** 10.3390/s19071682

**Published:** 2019-04-09

**Authors:** Jiamin Wu, Pan Guo, Sheng Shen, Yucheng He, Xin Huang, Zheng Xu

**Affiliations:** 1 State Key Laboratory of Power Transmission Equipment and System Security and New Technology, Chongqing University, Chongqing 400044, China; wujiamin@cqu.edu.cn (J.W.); shensheng@cqu.edu.cn (S.S.); heyucheng@cqu.edu.cn (Y.H.); xinhuang@cqu.edu.cn (X.H.); 2 School of Physics and Electronic Engineering, Chongqing Normal University, Chongqing 401331, China

**Keywords:** agriculture, inside-out nuclear magnetic resonance, constant gradient field, soil moisture

## Abstract

The improvement of water management in agriculture by exactly detecting moisture parameters of soil is crucial. To investigate this problem, a mini inside-out nuclear magnetic resonance sensor (NMR) was proposed to measure moisture parameters of model soils. This sensor combines three cylindrical magnets that are magnetized in the axial direction and three arc spiral coils of the same size in series. We calculated and optimized the magnet structure by equivalent magnetization to current density. By adjusting the radius and height between the cylinders, a circumferential symmetric constant gradient field (2.28 T/m) was obtained. The NMR sensor was set at 2.424 MHz to measure the water content of sandy soil with small particle diameter and silica sand with large particle diameter. The complete decaying, an NMR signal was analyzed through inverse Laplace transformation and averaged on a *T*_2_ space. According to the results, moisture content of the sample is positively correlated with the integral area of *T*_2_ spectrum peak (*A_peak_*); *T*_2_ of the water in small pores is shorter than that in large pores, because the movement of water molecules are limited by the inner wall of the pores. In the same volume, water in large pore sample is more than that in small pore sample, so *A_peak_* of silica sand is larger than *A_peak_* of sandy soil. Therefore, the sensor is capable of detecting moisture both content and pore size of the sample. This mini sensor (4.0 cm in diameter and 10 cm in length) is portable, and the lowest measurable humidity is 0.38%. Thus, this sensor will allow easy soil moisture measurements on-field in the future.

## 1. Introduction

The improvement of water management in agricultural and geological explorations by detecting moisture parameters of soil exactly is crucial. However, investigating such parameters is time-consuming and invasive in many cases. Thus, a fast and noninvasive approach is required to examine the moisture parameters of soils [1]. In the last few decades, nuclear magnetic resonance (NMR) has broadened its range of applications due to some new methods that use inhomogeneous static magnetic fields. The introduction of mobile NMR devices has been an important evidence that supports these new ideas. Inside-out NMR has found important new applications in the oil industry [2,3], hydrological studies [1,4], and medicine [5].

In the inside-out style of measurement, the measured object is outside of the magnet; that is, the magnetic field is open and, therefore, inhomogeneous. The natural measurement geometry is that of a borehole where the sensor may be displaced longitudinally through the sample. Given that boreholes have a circular cross section, the ideal sensitive volume for the inside-out NMR is toroidal in shape.

Numerous magnet designs and applications have been suggested for the inside-out NMR. Jackson [6,7] proposed two cylindrical magnets of the same size, with opposing poles (SN–NS), to produce a toroidal region of a static field external to the apparatus. A simple solenoid coil located between the magnets produced a radiofrequency (RF) field B_1_, orthogonal to B_0_ in the toroidal region. Taicher and Shtrikman [8] devised an inside-out NMR instrument in which a cylinder of nonconductive ferrite material was transversely magnetized, and the RF coil was wrapped along the longitudinal direction of the magnet. The resonance condition was satisfied in a thin cylindrical shell coaxial with the magnet. Kleinberg et al. [9] presented a device in which three slab magnets (two of the same size, with a third smaller magnet) were magnetized in the same direction and placed parallel with the small magnet in the center. This device provided a sensitive volume localized at a given sector with an axial extension. The antenna, which was essentially a half-coaxial cable, was placed in a semicircular cavity between the outer magnets.

In 2011, Sucre et al. [1] presented a new approach to measuring soil moisture. The sensor consisted of six cylindrical magnets (two end cylinders with a larger diameter and four centric cylinders with a smaller diameter that were magnetized perpendicular to the main axis), and a planar rectangle RF coil in the center of the magnet array. In 2013, the Sucre’s sensor was improved by Perlo et al. [10], using a five-turn rectangular RF coil with the wires placed at optimized angular positions. In this case, two cylindrical magnets with transverse polarization were utilized. The RF coil was placed in a groove on one side of the magnets. The sensitive volume in this sensor did not have an azimuthal symmetry.

Marble et al. [11] introduced a three-magnet array for single-sided NMR measurements. In this approach, three magnet blocks, which were magnetized in the same direction, were arranged to produce a homogeneous magnetic field. The magnetic field was parallel to the surface, and the design was naturally compact and safe. Even for the homogeneous magnet structure, the static field homogeneity was much less in mobile NMR devices than in conventional NMR experiments.

**Figure 1:**
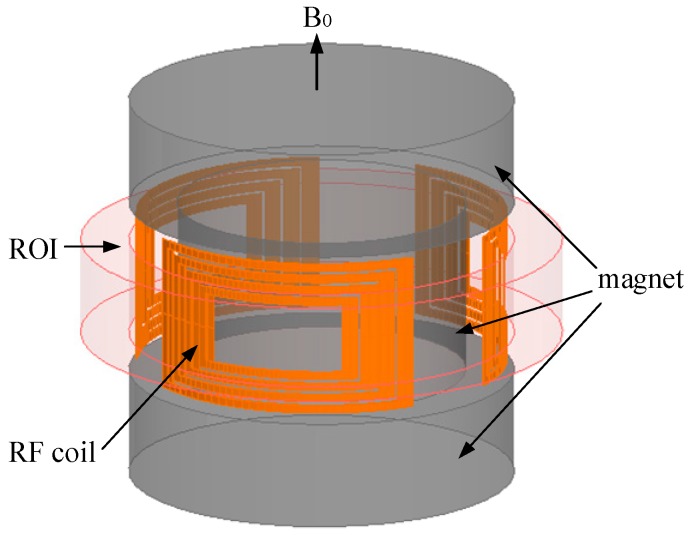
Mini NMR sensor model.

In this work, a mini inside-out sensor was proposed, as depicted in Figure 1. This approach combines the toroidal symmetry proposed by Jackson [7] with the advantages of the three-magnet array [11,12] concept to produce magnetic field with constant gradient in toroidal region of interest (ROI). Three arc spiral coils of the same size in series were used as the RF coil for excitation and detection. The mini NMR sensor was used to detect the moisture content of the sample that comprised soil and water. In subsequent sections, the design process of the magnet and RF coil are presented in detail, as are the experimental results.

## 2. NMR Sensor Design

### 2.1. Magnetic Field Calculation

**Figure 2:**
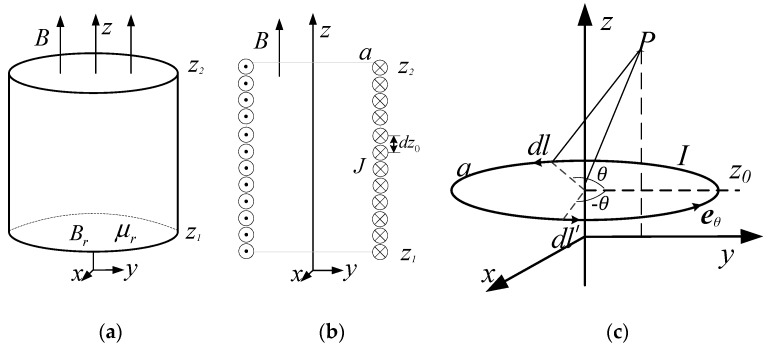
Calculation model of a cylindrical magnet (**a**). Approximation of a permanent magnet as the equivalent surface current density (**b**). Single-turn current for calculation (**c**).

We derive a simple analytical calculation method for the magnetic field produced by a cylindrical magnet. This magnet is magnetized along the z-direction and positioned with its upper surface at z_2_ and lower surface at z_1_, as illustrated in Figure 2a. The radius of the magnet is *a*, and the residual magnetization is *B*_r_. The magnet can be represented by an equivalent surface current distributed on the cylindrical surface. The direction of current and *B*_r_ satisfy the right-hand rule. The current can be divided into infinitesimal current elements of width dz_0_, as demonstrated in Figure 2b. The magnetic field induced by each current element, as exhibited in Figure 2c, can be calculated.

For a cylindrical magnet displayed in Figure 2a, equivalent surface current density ***J*** can be written as [13]

(1) $$J=\frac{B_{r}}{\mu_{0}}e_{\theta }$$

where *B_r_* is the residual magnetization, $\mu_{0}$ is the vacuum permeability, and $e_{\theta }$ is the unit vector in the circumferential direction.

The magnetic field induced by the current model presented in Figure 2c is derived by introducing the magnetic vector potential ***A*** (B=∇×A) and using Biot–Savart’s law. The ***A*** (at field point (ρ,θ,z) in the cylindrical coordinate system) only has a circumferential component given the circumferential symmetry. It can be expressed as

(2) $$A_{\theta }=\frac{\mu_{0}I}{\pi k}\sqrt{\frac{a}{\rho }}\left[\left(1-k^{2}\right)K\left(k\right)-E\left(k\right)\right]$$

where

$k^{2}=\frac{4a\rho }{\sqrt{{\left(a+\rho \right)}^{2}+{\left(z-z_{0}\right)}^{2}}}$, and *I* is the value of the current in the coil.
$K\left(k\right)=\int_{0}^{\frac{\pi }{2}}\frac{d\alpha }{\sqrt{1-k^{2}\sin^{2}\alpha }}$ is the first type of complete elliptic integral.
$E\left(k\right)=\int_{0}^{\frac{\pi }{2}}\sqrt{1-k^{2}\sin^{2}\alpha }d\alpha$ is the second type of complete elliptic integral.

On the basis of B=∇×A, we can obtain magnetic flux density ***B*** induced by a single-turn coil driven by a constant current *I*.

(3) $$B_{\rho }=\frac{\mu_{0}I}{2\pi }\frac{z}{\rho \sqrt{{\left(a+\rho \right)}^{2}+{\left(z-z_{0}\right)}^{2}}}[-K\left(k\right)+\frac{a^{2}+\rho^{2}+{\left(z-z_{0}\right)}^{2}}{{\left(a-\rho \right)}^{2}+{\left(z-z_{0}\right)}^{2}}E\left(k\right)]$$

(4) $$B_{z}=\frac{\mu_{0}I}{2\pi }\frac{1}{\sqrt{{\left(a+\rho \right)}^{2}+{\left(z-z_{0}\right)}^{2}}}\left[K\left(k\right)+\frac{a^{2}-\rho^{2}-{\left(z-z_{0}\right)}^{2}}{{\left(a-\rho \right)}^{2}+{\left(z-z_{0}\right)}^{2}}E\left(k\right)\right]$$

In Equations (3) and (4), *I* is replaced with $B_{r}/\mu_{0}\cdot dz_{0}$, and the variable *z*_0_ is considered integral in the interval [*z*_1_, *z*_2_]. The magnetic flux density ***B*** induced by the cylindrical magnet can be written as

(5) $$B_{\rho }=\frac{B_{r}}{2\pi }\int_{z_{1}}^{z_{2}}\frac{z}{\rho \sqrt{{\left(a+\rho \right)}^{2}+{\left(z-z_{0}\right)}^{2}}}\left[-K\left(k\right)+\frac{a^{2}+\rho^{2}+{\left(z-z_{0}\right)}^{2}}{{\left(a-\rho \right)}^{2}+{\left(z-z_{0}\right)}^{2}}E\left(k\right)\right]dz_{0}$$

(6) $$B_{z}=\frac{B_{r}}{2\pi }\int_{z_{1}}^{z_{2}}\frac{1}{\sqrt{{\left(a+\rho \right)}^{2}+{\left(z-z_{0}\right)}^{2}}}\left[K\left(k\right)+\frac{a^{2}-\rho^{2}-{\left(z-z_{0}\right)}^{2}}{{\left(a-\rho \right)}^{2}+{\left(z-z_{0}\right)}^{2}}E\left(k\right)\right]dz_{0}$$

### 2.2. Magnet Structure Optimization and Field Measurement

The construction of the magnet array of the mini NMR sensor is depicted in Figure 1. The sensor consists of three cylindrical magnets that are magnetized in the z-axis, and the diameter of the magnet is smaller in the center than at the ends to leave a certain space for the RF coil.

According to electromagnetic field theory, in the region near the magnet surface, two separated magnets produce an increasing field centered above and between them, whereas another magnet placed between them produces a decreasing field centered above [11,14]. The first or second derivatives of the static magnetic field in the ROI along the radial direction can be adjusted to 0 by properly adjusting the parameters of the three magnets (Figure 1). Homogeneous magnetic fields or magnetic fields with constant gradient can be induced using the three magnets. Thus, the parameters that must be optimized include the diameter and height of each magnet. Considering engineering applications, we limited the diameter and height of the sensor to at most 40 and 100 mm, respectively.

YXG32 Sm_2_(CoFeCuZr)_17_ cylindrical magnets (Ninggang Permanent Magnetic Materials Co., Ltd., Ningbo, Zhejiang, China) were applied, because this type of permanent magnet has a low temperature coefficient (−0.035%/°C) and high remanence (B_r_ 1.10–1.13 Tesla). Based on optimization, the central cylinder was 26 mm in diameter and 24 mm in length, whereas the two end cylinders were 30 mm in diameter and 10 mm in length. The titanium alloy housing used for fixing magnets has a diameter of 40 mm.

**Figure 3:**
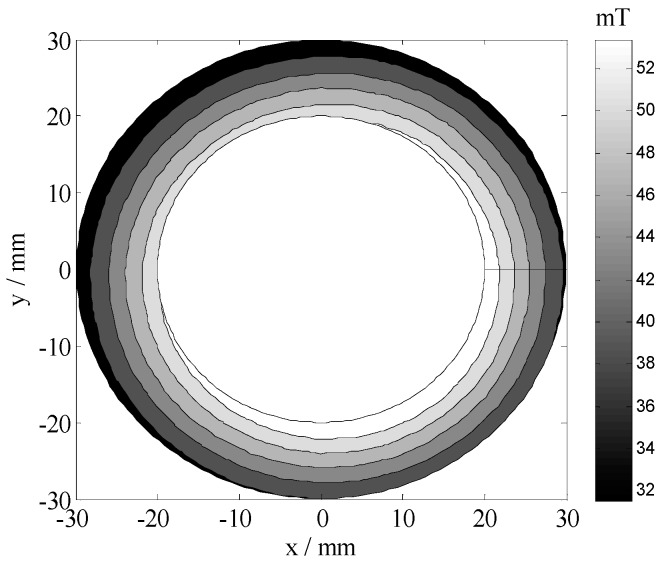
Magnetic field measured using a gauss meter in the ROI.

**Figure 4:**
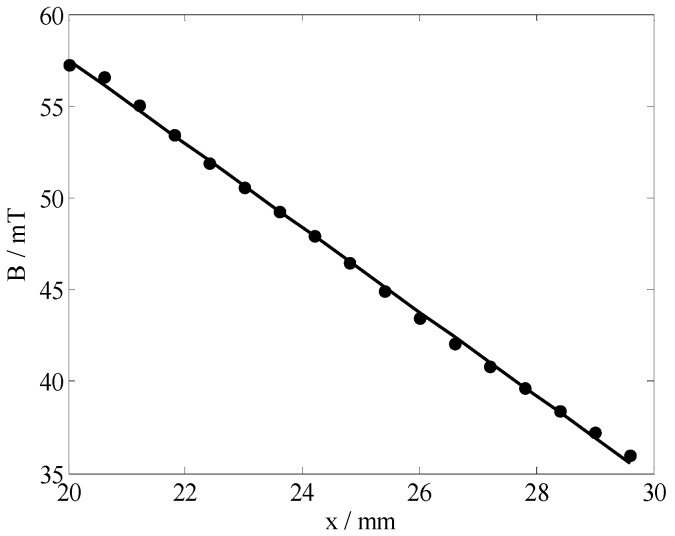
Magnitude of the magnetic field as a function of depth over the center of magnets.

The magnetic field was measured using a BELL 8030 Hall probe and a computer-controlled three-axis position system. Figure 3 illustrated a contour plot of the magnetic field magnitude around the magnet array. The position *ρ* = 20 (where *ρ*^2^ = *x*^2^ + *y*^2^) corresponds to the outside surface of the sensor. Slight asymmetry was noted given the unequal magnetizations of the magnets and measurement errors. However, the field in the ROI was reasonably symmetric. Figure 4 plots the magnitude of the magnetic field as a function of depth over the center of magnets. The approximately constant gradient at the region that occurs within 10 mm from the magnet surface corresponded to the ROI. The field gradient remains within the 2.28 T/m range over the region more than 10 mm deep.

### 2.3. RF Coil Design

**Figure 5:**
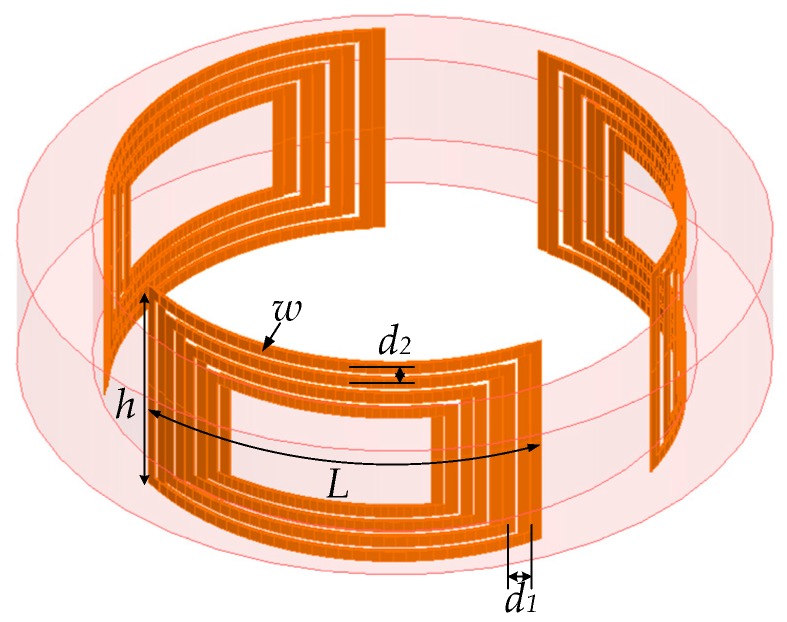
RF coil model. The arc length of each arc spiral coil with *N* turns is *L*, the width of wire is *w*, the height of wire is *h*, and the spacing of the adjacent wires along the circumference and *z*-direction is *d*_1_ and *d*_2_, correspondingly.

To excite hydrogen and receive as many signals as possible in the ROI, three arc spiral coils of the same size connected in series (Figure 5) were used as the RF coil. Based on reference [15], when the experimental environment and the *B*_0_ remained the same, the SNR was proportional to the *B*_1_ generated by the RF coil and inversely proportional to the current through the RF coil and the square root of the AC resistance of the coil, as in Equation (7).

(7) $$\mathrm{SNR}\propto \frac{B_{1}/i}{\sqrt{R}}\cdot V_{\mathrm{sample}}$$

where *V*_sample_ is the effective sample volume; *R* is the combined noise resistance of coil, sample, and spectrometer electronics; and *B*_1_/*i* is the coil sensitivity defined by the law of reciprocity as the magnetic flux density *B*_1_ caused by an RF current *i* that passes through the coil. We defined the right side of Equation (7) as the relative SNR for evaluating the RF coil.

**Figure 6:**
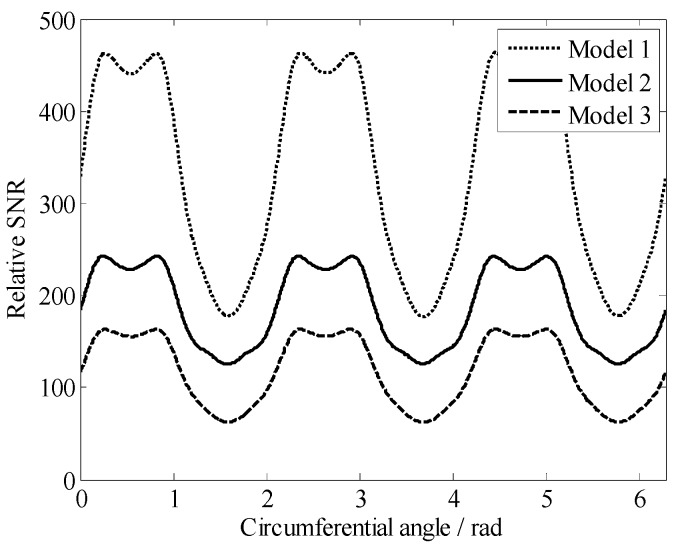
Relative SNRs of the RF coils with different structure parameters, where, parameters of Model 1 are *N* = 4, *L* = 28 mm, *h*=15.2 mm, *w* = 0.8 mm, *d*_1_ = 2 mm, and *d*_2_ = 1.2 mm; parameters of Model 2 are *N* = 6, *L* = 32.3 mm, *h*=15.2 mm, *w* = 0.6 mm, *d*_1_ = 1.5 mm, and *d*_2_ = 0.9 mm; and parameters of Model 3 are *N* = 6, *L* = 34.3 mm, *h*=15.2 mm, *w* = 0.6 mm, *d*_1_ = 1.5 mm, and *d*_2_ = 0.9 mm.

To facilitate the installation of the RF coil, we printed it on a flexible printed circuit board (fPCB). The effective value of the AC current was set to 1 A at 2 MHz, and the values of *B*_1_ produced by the RF coil and *R* were calculated using the simulation software Maxwell 3D (ANSYS, Inc., Canonsburg, PA, USA). By varying the parameters of the RF coil, the optimal RF coil was limited by the size of the magnet body and shell, the wring area of the arc spiral coil achieved relatively homogeneous excitation and signal reception across the sensitive volume. The parameters of RF coils that must be optimized include the turns of the coil, the distance between adjacent wires, and the width of wires. We simulated dozens of coils, and Figure 6 demonstrates the results of three of these models. The optimal coil is Model 1. Finally, this mini sensor is 40 mm in diameter and 100 mm in length and is portable for soil moisture measurements on-field.

## 3. Results and Discussion

**Figure 7:**
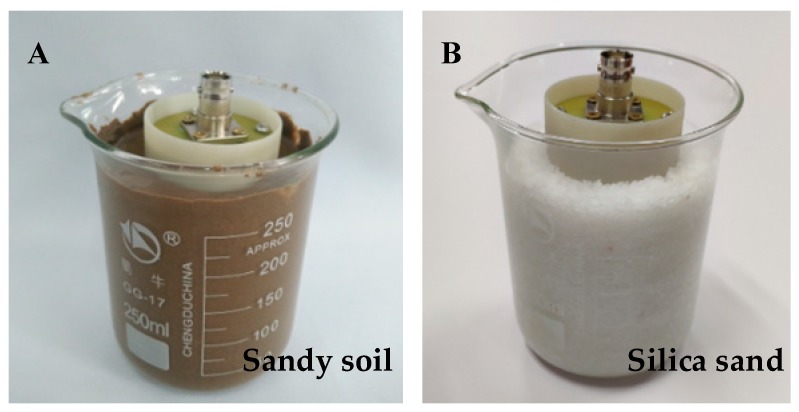
Sandy soil and silica sand.

In this work, we selected sandy soil and silica sand (shown in Figure 7) with water to prepare an experimental sample, and then we measured them through Carr–Purcell–Meiboom–Gill (CPMG) measurements and weighing analysis to determine the moisture content of this sample. The moisture content *η* could be determined through the weighing method.

(8) $$\eta =\frac{m_{1}-m_{2}}{m_{1}}\times 100\%$$

where *m*_1_ is the weight of the sample with water, and *m*_2_ is the weight of the sample without water, the weight is measured by an electronic balance (precision is 0.001 g).

Sample A is a mixture of sandy soil (219.613 g in weight, particle diameter is smaller than 0.25 mm) and water (38.538 g in weight), the initial moisture content is 17.55%. Sample B is a mixture of silica sand (216.367 g in weight, particle diameter is between 0.85 mm and 2 mm) and water (62.004 g in weight), the initial moisture content is 22.27%. To measure the samples with different moisture contents, we dried it continuously in a humidity chamber. The temperature in the humidity chamber was 40 °C for drying the samples.

**Figure 8:**
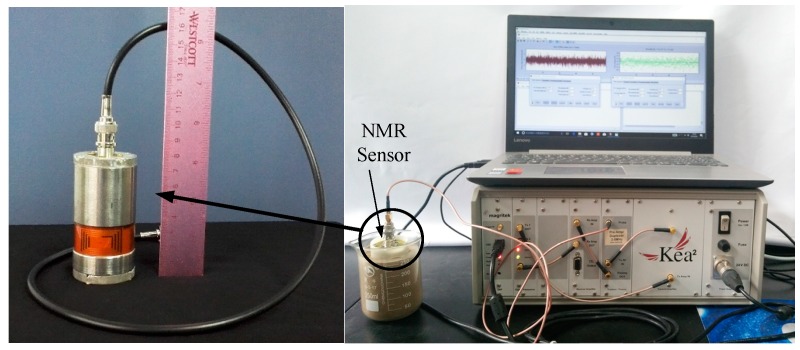
Measurement system.

**Figure 9:**
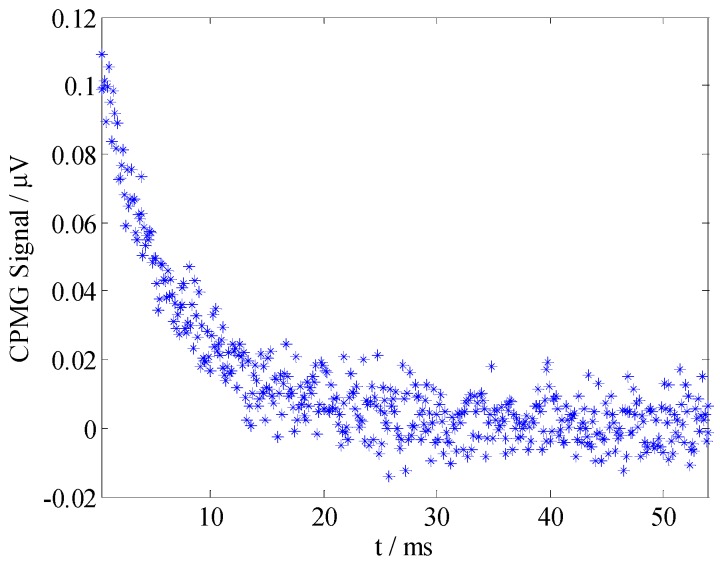
T_2_ decay measured using a CPMG sequence for sample A (moisture content is 8.53%).

The mini sensor was set at a certain depth to measure the water content of the soil sample. Experiments were conducted through a Magritek Kea2 Spectroscopy (Wellington, New Zealand), which consists of an internal preamplifier and lumped element duplexer. The measurement system is depicted in Figure 8. A total of 2100 echoes from the sample were obtained in 2048 scans through the CPMG experiment; the 90° and 180° pulse widths were 12 μs, the NMR frequency was 2.424 MHz, and the echo time was 120 μs. Figure 9 displays a CPMG decay measured using 2048 signal sums. The complete decaying NMR signal is analyzed through inverse Laplace transformation and averaged on a T_2_ space.

The CPMG decays are analyzed in terms of multiple exponential fitting [16]

(9) $$y(t)=\sum_{j=1}^{m}f(T_{2j})e^{-t/T_{2j}}+\xi (t),$$

where *y*(*t*) is amplitude of CPMG signal at *t*, *f*(*T*_2*j*_) is the amplitude of component with transverse relaxation time *T*_2*j*_, *ξ*(*t*) is stochastic noise, *m* is the number of relaxation components, and *T*_2_ spectrum curve is the relationship between *T*_2_ with *f*(*T*_2_). The integral area (*A_peak_*) of peak in *T*_2_ spectrum curve can be expressed as

(10) $$A_{peak}=\int_{T_{2\min}}^{T_{2\max}}f(T_{2})dT_{2},$$

**Figure 10:**
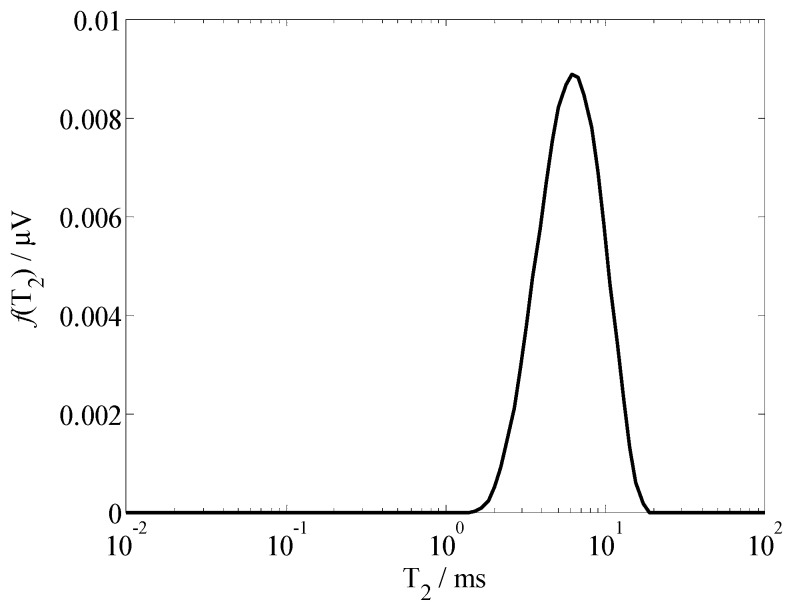
*T*_2_ spectrum curves of sample A (moisture content is 8.53%).

Thus, by substituting the measured data into Equation (9), we can obtain the *T*_2_ spectrum *f*(*T*_2_). The *T*_2_ spectrum of sample A (moisture content is 8.53%) is presented in Figure 10. To verify performance of the sensor, we measured sample A (moisture content is 8.53%) five times, *A_peak_* are 0.8904, 0.8759, 0.8636, 0.8675, and 0.8384, respectively; averaging at 0.8672, the maximum deviation ratio ($\left|A_{peak}-{\bar{A}}_{peak}\right|/{\bar{A}}_{peak}\times 100\%$) is 3.32%.

**Figure 11:**
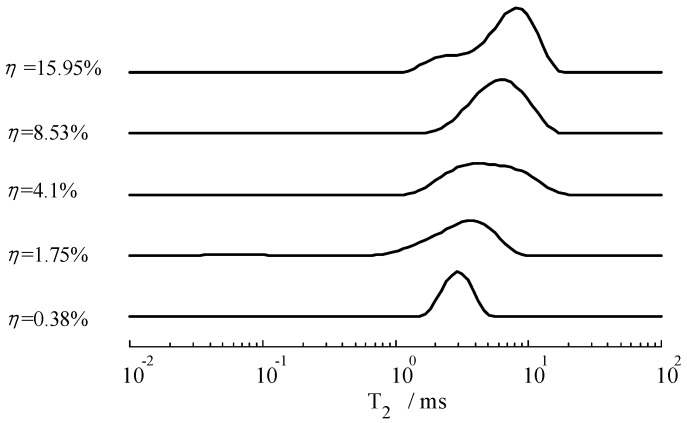
*T*_2_ spectrum curves of the sandy soil with different moisture content.

**Figure 12:**
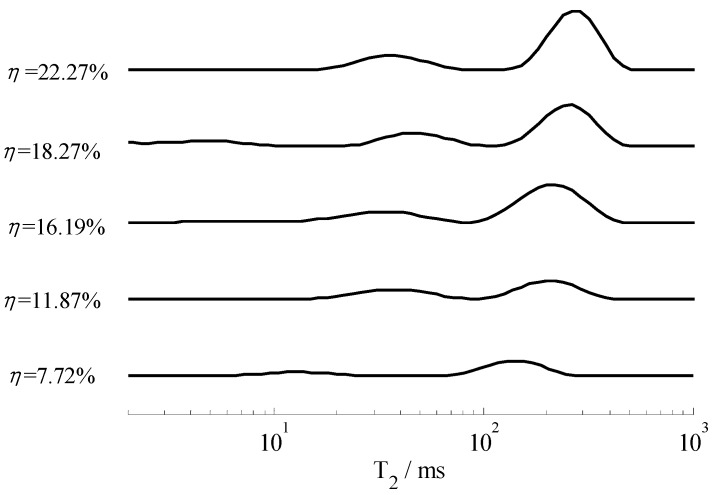
*T*_2_ spectrum curves of the silica sand with different moisture content.

**Figure 13:**
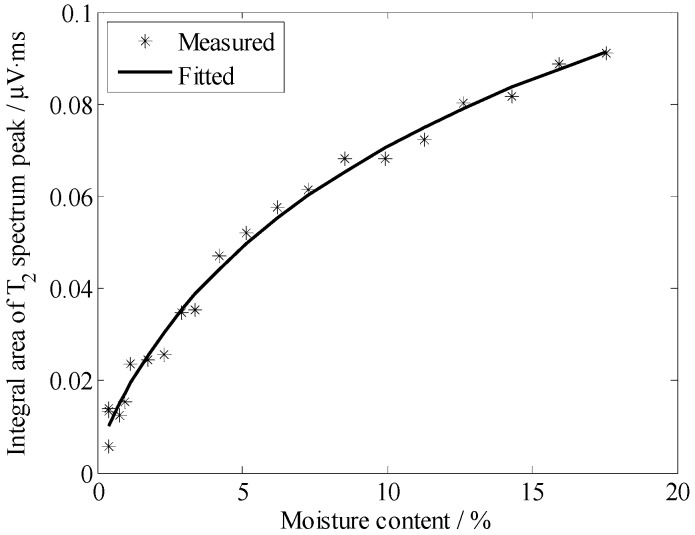
Correspondence between the integral area of the *T*_2_ spectrum with the moisture content of the sandy soil.

**Figure 14:**
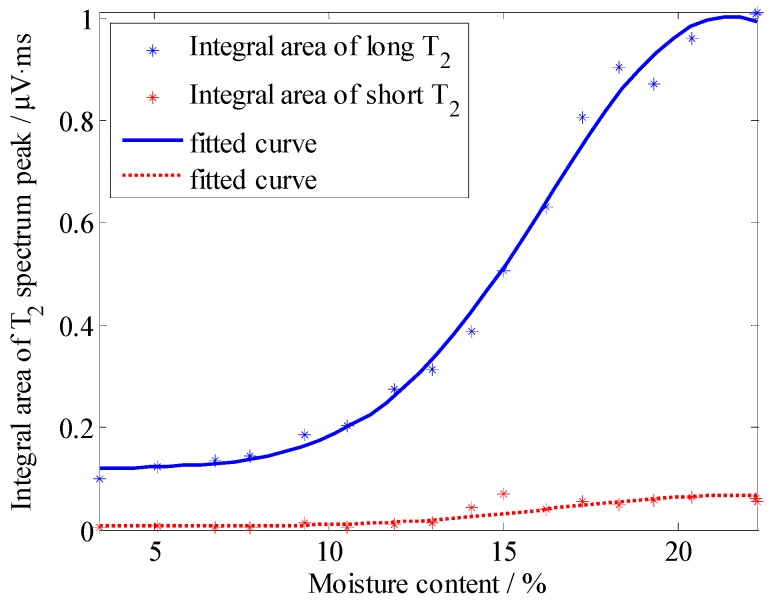
Correspondence between the integral area of the *T*_2_ spectrum with the moisture content of the silica sand.

Considering graph visualization, Figure 11 and Figure 12 only illustrates five results of different moisture contents, although we measured the samples every hour throughout the day. According to Figure 11 and Figure 12, we know that the position of the *T*_2_ spectrum peak shifted to the left gradually with the decrease of moisture. To further study the relationship between moisture content and *T*_2_ spectrum, we calculated the integral area of each spectrum peak (*A_peak_*), then graph the relationship between *A_peak_* with *T*_2_, illustrated in Figure 13 and Figure 14. These curves could indicate the integral area of the *T*_2_ spectrum, whichis positively correlated with moisture content.

According to Figure 11 and Figure 12, the *T*_2_ spectrum curves of the sandy soil only have one peak, whereas the *T*_2_ spectrum curves of the silica sand have two peaks (a long *T*_2_ peak and a short *T*_2_ peak). The reason for this phenomenon is that particle diameter of sandy soil is almost the same size and the pore size is basically the same, while particle diameter of silica sand is 0.85~2 mm and sample include large pores and small pores. *T*_2_ of the water in small pores is shorter than that in large pores, because the movement of water molecules are limited by inner wall of the pores. In the same volume, there is more water in the large pore sample is than in the small pore sample, so *A_peak_* of silica sand is larger than *A_peak_* of sandy soil. In Figure 13 and Figure 14, the results indicate that *A_peak_* of the large pore sample (silica sand) is more sensitive to represent the changes in moisture than that of the small pore sample (sandy soil) when moisture content is high, and *A_peak_* of the small pore sample (sandy soil) is more sensitive to represent the changes in moisture than that of the large pore sample (silica sand) when moisture content is low.

## 4. Conclusions

In this work, we presented a mini NMR sensor that combines three cylindrical magnets to measure moisture content effectively in sandy soil and silica sand. The sensor has a constant gradient of 2.28 T/m. A novel RF coil composed of three arc spiral coils of the same size connected in series was designed for inside-out NMR applications. The coil demonstrated a high SNR, mostly due to an enhancement in the sensitivity region. The complete decaying NMR signal was analyzed through inverse Laplace transformation on the *T*_2_ space, which could estimate the moisture content of the samples. This mini sensor is portable, and the sensitive region is relatively larger than its actual size. In conclusion, this mini NMR sensor has been demonstrated to be a fast and a noninvasive technique for investigating the moisture content of soils. Moreover, this mini sensor may allow easy on-field soil moisture measurements in the future. Further research should focus on applying this sensor to pore media measurement, thereby improving stability and detection depth by further optimizing the sensor structure.
